# A Missed Opportunity? How Health Care Organizations Engage Primary Care Clinicians in Formal Social Care Efforts

**DOI:** 10.1089/pop.2021.0306

**Published:** 2022-08-08

**Authors:** Taressa K. Fraze, Laura B. Beidler, Laura M. Gottlieb

**Affiliations:** ^1^ Department of Family and Community Medicine, University of California San Francisco, San Francisco, California, USA.; ^2^ Healthforce Center, University of California San Francisco, San Francisco, California, USA.; ^3^ Philip R. Lee Institute for Health Policy Studies, University of California San Francisco, San Francisco, California, USA.; ^4^ The Dartmouth Institute for Health Policy and Clinical Practice, Geisel School of Medicine, Lebanon, New Hampshire, USA.

**Keywords:** social care, social determinants of health, primary care, clinicians, workforce

## Abstract

Health care organizations increasingly recognize the impact of social needs on health outcomes. As organizations develop and scale efforts to address social needs, little is known about the optimal role for clinicians in providing social care. In this study, the authors aimed to understand how health care organizations involve clinicians in formal social care efforts. In 2019, the authors conducted 33 semi-structured interviews with administrators at 29 health care organizations. Interviews focused on the development and implementation of formal social care programs within the health care organization and the role of clinicians within those programs. A few administrators described formal roles for primary care clinicians in organizational efforts to deliver social care. Administrators frequently described programs that were deliberately structured to shield clinicians (eg, clinicians were not expected to review social risk screening results or be involved in addressing social needs). The authors identified 4 ways that administrators felt clinicians could meaningfully engage in social care programs: (1) discuss social risks to strengthen relationships with patients; (2) adjust clinical care follow-up plans based on social risks; (3) modify prescriptions based on social risks; and (4) refer patients to other care team members who can directly assist with social risks. Administrators were hesitant to increase primary care clinicians' responsibilities by tasking them with social care activities. Defining appropriate and scalable roles for clinicians along with adequate support from other care team members may increase the effectiveness of social care programs.

## Introduction

Consistent and compelling evidence shows that social factors, including food, housing, and financial security, impact health outcomes.^1,2^ Social, economic, and behavioral factors are estimated to drive up to 80% of health outcomes. This evidence has compelled health care organizations to invest in social care activities.^1^ Further, recognizing these links, the National Academies of Sciences, Engineering, and Medicine (NASEM) recently recommended 5 ways in which social care could be integrated into medical care delivery with the goals of improving patients' social conditions and health outcomes.^3^

This included activities to increase awareness of social risk factors; adjust care plans according to social risks; assist patients in improving their social conditions; participate in activities that align needs with community resources; and advocate for policies that address social needs.^3^ Prior research has already shown that primary care settings are transforming care delivery to include social care activities such as screening patients for social risks, providing patients with referrals to community resources, and assisting patients access resources.^4^

Despite increases in social risk screening, little is known about how health care organizations engage clinicians in their formal efforts to integrate social care into medical care. Suggestions about potential roles for clinicians in the delivery of social care have included acting as champions for social care programs, integrating information on patients' social risks in routine clinical encounters by adapting care plans, and even actively engaging in coordinating medical and social care across sectors.^5,6^ However, a significant barrier is that clinicians typically receive little to no training on how to integrate social care into medical care.^6,7^ Health care organizations may be hesitant to formally incorporate clinicians in social care efforts if they are uncertain how clinicians can best help patients or if clinicians face competing demands.^8,9^

Despite calls for clinicians to assume an active role in social care delivery, little is known about the roles of clinicians in the health care organization's efforts to deliver social care. In this study, the authors interviewed administrators at health care organizations to learn about how and why clinicians are involved in formal social care efforts, including their roles, responsibilities, and involvement in decision making.

## Methods

In 2019, the authors conducted 33 semi-structured interviews with administrators at 29 health care organizations about their efforts to identify and address patients' social needs.^10,11^ The goal of this study was to understand formal, organizational approaches to delivering social care such as social risk screening, social needs referrals, and social care in case management. For these analyses, the authors focused on how clinicians are involved in formal social care efforts within health care organizations. This study was approved by Dartmouth College Committee on the Protection of Human Subjects.

### Sample

Organizations were selected because they had active social care programs. The authors identified organizations using 2 methods. First, the authors identified potential organizations from those that responded on the National Survey of Health Care Organizations (NSHOS)^4,12–15^ that they screened patients for 5 key social risks (food insecurity, housing instability, transportation, utilities, and interpersonal violence).^12,16^ The NSHOS is a nationally representative suite of surveys that included a system-level survey (*N* = 325, response rate = 57%) and a practice-level survey (*N* = 2190; response rate = 44%).^12^ Second, the authors searched the Internet for publicly available information (eg, news articles and press releases) to identify health care organizations with active social care programs.

The authors emailed leaders at sampled organizations and asked them to connect the research team with the individual at their organization who was best suited to speak about their social care.

Interviewed individuals, identified by leaders in each organization, had varying titles (Supplementary Appendix Table SA1). However, all interviewed individuals were responsible for administering social care within their organizations (hereafter referred to as administrators). The authors contacted 34 organizations identified via NSHOS and 30 organizations identified via Internet searching (Supplementary Appendix Table SA2). The authors conducted interviews with 29 organizations that agreed to participate (11 from the NSOHS sample and 18 from the web-based sample).

At 4 organizations, the authors conducted a second interview to gain additional information about the organization's social care efforts. Supplementary Appendix Table SA4 provides specific reasons for each secondary interview. Organizations were diverse in size (single provider practice to multi-state health systems), geography, and ownership (Supplementary Appendix Tables SA2–SA4). Outreach was conducted until achieving a point of saturation, with no new themes uncovered during interviews.^17^

### Interview guide and data collection

Interviews followed a semi-structured interview guide that included questions about: (1) organizational structure; (2) approaches to screening for social risks; (3) referrals to community-based organizations; (4) approaches to assisting patients with social needs; and (5) interactions with community-based organizations. Across each of these domains, interviewers probed on the role of clinicians in formal program development and implementation (Supplementary Appendix Table SA5). Interviews lasted ∼60 minutes and were conducted by trained qualitative researchers (T.K.F. and L.B.B.) via telephone. Interviews were recorded once participants provided informed consent and then professionally transcribed.

### Analysis

The authors used a grounded theory approach to guide their analysis. Grounded theory is both a flexible and complex approach that includes purposive sample, coding, constant comparative analysis, and formal memoing.^18^ The authors first conducted broad initial coding by using NVivo.^19^ This coding was conducted by a trained qualitative researcher (L.B.B.) or a trained research assistant. Initial codes were aligned with domains of the interview guide (Supplementary Appendix Table SA5).^20^ Intercoder reliability was established through iterative double coding of transcripts by coders and lead author (T.K.F.) until all agreed (Supplementary Appendix Fig. SA1).^21^

To gain a more nuanced understanding of the data, the authors conducted intermediate coding on the involvement of clinicians in formal social care programs.^18^ One team member (L.B.B.) iteratively sub-coded the transcripts, and the lead author (T.K.F.) reviewed all sub-coding. Two authors (L.B.B. and T.K.F.) met weekly to discuss coding and resolve any disagreements. To organize and support analyses, 2 authors (L.B.B. and T.K.F.) iteratively developed an analytic memo that described all observed themes and recorded how each organization fit within each theme.^20^ To demonstrate the validity and trustworthiness of the data, the authors used a matrix coding approach that justified the inclusion or exclusion of each organization within each theme.^20^

## Results

Interviewed organizations were diverse in terms of size, geography, and ownership (Supplementary Appendix Table SA2). All administrators described organizational efforts aimed at identifying and addressing patients' social needs within primary care settings. Typical social care activities included providing referrals to community-based organizations, assistance accessing resources, and regular follow-up with patients to provide additional support.

A few administrators described clinicians as being directly or substantively involved in formal organizational efforts to address patients' social needs (Table 1). Administrators expressed concerns around “burdening” clinicians and designed programs that *purposefully* limited clinician involvement due to significant existing responsibilities. As 1 administrator explained, “It's yet one more thing that they've [clinicians] got to add to the encounter.”

**Table 1.** Spectrum of Clinician Engagement in Organizations' Formal Social Care Programs

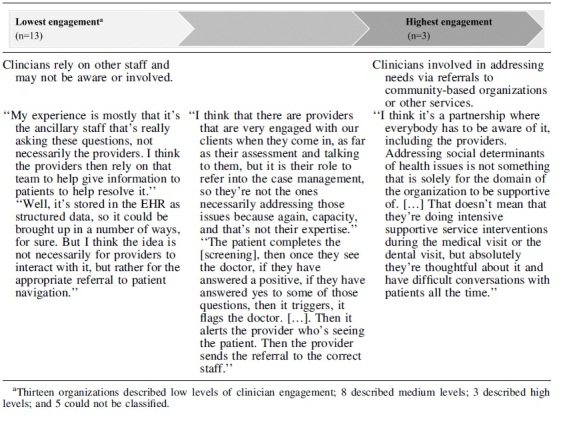

### Spectrum of clinician involvement in social care

Clinician involvement ranged along a spectrum from no formal involvement or awareness to clinicians leading and directing social care efforts (Table 1). On 1 end, most administrators reported that clinicians were not at all involved in *formal* organizational efforts to address social needs, that social needs screening data were not easily accessible via the electronic health record or that there were deliberate efforts to shield clinicians from additional work (*n* = 13). One administrator noted that clinicians could interact with screening data, but it was not part of the standard workflow: “So the [social needs screening] information is in the medical record that people could have access to it whether or not they look for it. There's no system around that necessarily.”

On the other end of the spectrum, clinicians assumed a greater responsibility in social care programs. These programs were uncommon (*n* = 3) and represented a significant investment of clinician effort. For example, 1 physician leader at a small, rural practice explained how he and his clinical team were deeply invested in whole-person care for their patients. They routinely coordinated with families, other medical providers, and social services. One key factor that distinguished these programs from programs with lower clinician involvement was that the programs were spearheaded by the clinicians involved.

Unlike lower intensity programs, these programs were driven by personal interests of clinicians rather than developed by health care administrators, which highlights the importance of having a clinical champion if clinicians are expected to assume greater responsibility for social care.

The remaining administrators described their clinicians' roles as somewhere between the 2 ends of the spectrum. Administrators wanted clinicians to be aware of a patient's social needs and to consider those needs when making a care plan, but they did not expect clinicians to be involved in directly addressing needs. In these programs, other members of the care team, such as social workers or community health workers, assisted the patient. One community health worker described her interactions with clinicians:

They might come up and say, “Hey [name], I have something for you to do. Can you check into this for this person? Can you check into transportation for this person? They're having a hard time finding food resources, can you help?” […] So once a week, case management, CHAs, physicians, front office people, we have a meeting once a week called a huddle. Where we're all together and we discuss patients and priorities.

### Roles for clinicians in delivering social care

Administrators focused formal social care efforts on activities that could be implemented by other, non-clinician care team members in an effort to reduce clinician burden. Social care was often described as a team effort: “It may not become central to the provider's discussion, but it's certainly a part of a more care team approach to a patient.” Despite this emphasis on non-clinician team members, administrators raised 4 ways in which organizations could engage clinicians to use information on patients' social risks in routine clinical encounters (Table 2).

**Table 2.** Potential Clinician Roles in Formal Organizational Activities to Integrate Clinical and Social Care

Role	Quotes^a^	Potential actions for clinicians within formal programs^b^
Strengthen relationships with patients	“This physician started asking these questions as part of the annual wellness visit and so we asked him about that and he said, ‘I don't feel like I need to resolve everyone's positive SDOH questions. It leads to a conversation, and it's about the patient provider conversation. When someone responds positively about social isolation, then I can talk with them about it. I don't need to fix it for them.’”	• Encourage patients to feel comfortable sharing concerns regarding social needs. • Have discussions with patients around contextual challenges.
Adjust health care follow-up schedules	“If we can't get the transportation arranged, maybe we ought to think about some alternative like should we connect them to our telehealth service? Or should we be thinking about how to get them somebody to go to their home rather than requiring that they come back in seven days to see their primary care provider, or maybe we need a home health nurse to go out and take their stitches out. […].”	• Change the follow-up timeline. • Consider accessibility of specialists when making referrals (eg, on a bus route). • Consider telehealth. • Offer a home visit.
Modify prescriptions	“When you're thinking about the medicine that you prescribe, you have to understand whether your patient can afford it or not, and are they going to fill the prescription or are they just going to smile and nod at you? Are they going to take it according to the way that you ask them to, or are they going to cut the pills in half? Are you prescribing something that they're going to have on them that has street value where they're at risk of being assaulted or robbed because you're giving them medicine that somebody is going to want to steal and take from them? Are they in a place where they are safe?”	• Consider cost of prescriptions. • Consider if the patient can easily store the medication (eg, access to refrigerator for insulin). • Consider the impact of side effects (eg, a diuretic for a homeless patient).
Refer patients to other care team members	“What I expect is that it's going to be really fluid between both the front desk as well as the care team, that if something comes up in the clinician encounter or the medical encounter, they certainly could, if the person [case management] is available, just do a warm handoff and take care of their needs then.”	• Make sure patients know why this is important to their health. • Encourage patients to meet with other care team members.

^a^ Some organizations described specific examples of how clinicians in their organizations integrated social and clinical care whereas other organizations described how they envisioned clinicians might deliver social care.

^b^ Potential actions are based on suggestions from interviewees and expanded by

First, administrators suggested that clinicians could more systematically use information on social factors to strengthen relationships with patients. Knowing more about their patients' lives enables clinicians to build trust and engage meaningfully with patients. As 1 administrator described, “That doesn't mean that they're doing intensive supportive service interventions during the medical visit or the dental visit, but absolutely they're thoughtful about it and have difficult conversations with patients all the time. That's just part of being a caring provider.”

Second, administrators suggested that clinicians could schedule follow-up visits to accommodate patients' social needs. For example, if a patient struggles with transportation to appointments, a clinician can modify the visit frequency, schedule visits when the patient can access transportation, or offer telehealth.

Third, clinicians could factor in a patient's social risks when prescribing medications, which might increase medication adherence. For example, they could discuss the potential side effects of a prescription (eg, increased urination) or a patient's living condition (eg, no reliable access to a bathroom) with the patient when developing a treatment plan. One administrator explained that costs should be factored into prescribing by saying, “Don't prescribe them a medicine that you know is $120 to pick up. Can you get them in a patient assistance program? Does it need a prior authorization? What are the steps for that?”

Finally, clinicians could refer patients to other care team members who can assist patients with social needs. Some administrators noted that other team members (social workers, community health workers, care managers, etc.) were trained to assist patients with social needs and clinicians could refer to these team members. One administrator, who was also a practicing clinician, described how conversations with patients can be beneficial and emphasized the importance of introducing patients to other care team members:

I don't envision the primary care physician being able to see a screen in the chart and then personally themselves address all of these issues. I think it makes sense for them to be aware of them and have **a brief conversation with the patient about them** and help, ideally, be able to work on some kind of **warm hand off with case managers** or community health workers or social workers, or whomever they have available on their team, to start addressing those issues.

Despite this breadth of approaches for how clinicians *could* be involved, administrators typically felt that, at most, clinicians *should be formally expected* to refer patients to other care team members.

## Discussion

Health care administrators recognized the impact of social circumstances on health and developed organizational-level programs to assist patients with social needs. Programs were strategically designed to maximize the efforts of the broader care teams while minimizing the impact on clinicians: Nearly half indicated that clinicians were *not* involved in formal social care programs. Instead, formal organizational efforts to deliver social care were often siloed with other care team members responsible for implementing activities.

Despite limited clinician involvement in formal social care efforts, administrators felt that clinicians could have a role in addressing social needs and identified ways that clinicians could become more involved in social care delivery, including by strengthening relationships with patients, adjusting follow-up schedules, modifying prescriptions, and referring patients to other care team members.

Existing pressures on primary care teams likely influenced the finding that most interviewed health care administrators consciously limited the role of clinicians in formal organizational efforts to deliver social care. These pressures stem from a limited clinician workforce,^22^ a graying population with complex clinical needs,^23,24^ chronic underfunding,^25^ and health care delivery reform.^26^

Other research has underscored that, when possible, health care leaders rely on non-clinician care team members, such as care managers, to implement care delivery innovations.^27–30^ The current study aligns with findings from prior research that found that although clinicians supported screening for social risks, most did not feel confident in their ability to address identified needs.^31^ Clinicians were particularly concerned about a lack of time, resources available, and training as barriers to delivering social care.^31^ Additional studies have shown that a clinician's perceived inability to address patients' social risks is linked with higher rates of burnout,^8,32^ aligning with the current findings that administrators may attempt to shield clinicians from efforts to address social needs.

Although clinicians are often viewed as integral for successful implementation of care delivery reforms, especially around building and sustaining buy-in for change,^33,34^ this study found that health care administrators were strategic in how they engaged with clinicians and built buy-in. The integration of social care into medical care could represent a substantial change to care delivery; thus, requiring full clinician engagement could limit uptake, underscoring why administrators may engage other team members in these formal programs. The authors' findings underscore the increasingly critical role that non-clinician care team members play in care delivery transformation, including social care efforts.

Although it is understandable that health care administrators may be hesitant to increase clinician responsibilities, this approach may also miss meaningful opportunities for clinicians to deliver social care. Instead of deliberately shielding clinicians from formal social care efforts, administrators could strategically engage clinicians by focusing their efforts within formal programs. As administrators consider how to integrate clinicians into social care efforts, additional training will likely be needed to ensure clinicians are confident in their skills.

Interviewed administrators discussed their ideas for how to best involve clinicians in formal social care efforts. These suggestions aligned with types of care included in the NASEM 2019 report.^3^ Two of the 4 roles envisioned for clinicians could likely be implemented in clinical settings, with minimal additional responsibilities placed on clinicians.

First, increased awareness of social conditions can be used to strengthen patient–provider relationships and lead to increased patient engagement and retention.^35,36^ Second, encouraging patients to engage with other care team members may help patients feel more comfortable accessing assistance.^37^ Care team members, such as community health workers, care managers, or social workers, play an increasingly pivotal role in primary care delivery by coaching patients to achieve their health goals, coordinating services, liaising with community-based organizations, and regularly following up with patients.^38–41^

The other 2 roles envisioned for clinicians, adjusting care plans through follow-up schedules and prescribing, may be more operationally challenging to implement within routine encounters. Adjusting prescriptions may require a more in-depth comparison of potential side effects with the patient's social circumstances or an understanding of the patient's costs associated with medications.

Recognizing that adjusting care plans to accommodate social risk factors may improve health outcomes through increased patient adherence,^42^ health care organizations could develop routine processes that facilitate social care activities. New technologies may also facilitate social care. Social risk screening could be seamlessly integrated into electronic health records so that clinicians have easy access to screening results.^43^

Social risk factors could then be integrated into standardized clinical decision tools; for instance, when a patient screens positive for transportation barriers, clinicians could be prompted to consider if telehealth is appropriate for follow-up visits or patients could be referred to care management for support accessing services. Real-time pharmacy benefit tools may enable clinicians to engage in conversations around costs.

Although technology-based solutions may ease the burden of the clinician's social care activities, there will likely still be significant implementation challenges. Further, technology-based solutions, though on the horizon, may still be a way off for many clinicians. In the meantime, care management teams could likely support efforts to adjust care plans for patients with social risks through activities such as enhanced medication reconciliation (ie, how do social risks interact with medication side effects) and coordinating with clinicians to adjust follow-up schedules or referrals. Along with the primary care team, pharmacists can support social care activities by also ensuring prescriptions are aligned with patients' social risks.

This study has 2 key limitations. As a qualitative study, these results are not meant to be generalized to all health care organizations. These findings should be used to provide a context to advance research and policy related to primary care clinicians' formal roles in delivering social care. In addition, interviewees were primarily in management roles and may not be aware of all clinician activities to deliver social care, particularly those that occurred outside of formal programs.

Administrators in health care organizations are often responsible for spearheading the development and implementation of care delivery transformation activities, including social care. As such, administrators lend an important and insightful view into how clinicians are engaged in social care programs.

## Conclusions

Health care organizations may be missing meaningful opportunities to deliver high-value care because of their reticence to increase clinicians' responsibilities. The Centers for Medicare and Medicaid Services (CMS) have emphasized that high-value primary care should advances equity and improve social conditions.^44^ Thus, engaging clinicians in social care activities may help primary care practices effectively participate in medical homes, advanced primary care efforts, or accountable care arrangements.

Health care organizations can strategically diffuse social care responsibilities across the broader care team to ensure patients' care plans are tailored to their social risks while also managing clinician (and other care team members') burden.

## Supplementary Material

Supplemental data

Supplemental data

Supplemental data

Supplemental data

## Supplementary Material

Supplementary Appendix Figure SA1

Supplementary Appendix Table SA1

Supplementary Appendix Table SA2

Supplementary Appendix Table SA3

Supplementary Appendix Table SA4

Supplementary Appendix Table SA5
