# Supplementary material for: A Missed Opportunity? How Health Care Organizations Engage Primary Care Clinicians in Formal Social Care Efforts
Source: Popul Health Manag. 2022 Aug 8;25(4):509–16. doi: 10.1089/pop.2021.0306 (PMC9419929; doi:10.1089/pop.2021.0306)
Supplement: Supplemental data [file Suppl_AppendixTableS2-S4.docx]

Appendix Table 2: Characteristics of all invited organizations

| **Site** | **Organization type** | **Region** | **Safety net practice**** | **Primary care only***** | **Practice**  **urbanicity***** | **Outreach Wave*** | **Participate in interview?** |
| --- | --- | --- | --- | --- | --- | --- | --- |
| 1 | Practice | Midwest | No | Yes | Urban | 1 | Yes |
| 2 | Health system | Northeast |  |  |  | 1 | Yes |
| 3 | Practice | West | Yes | Yes | Multiple | 1 | Yes |
| 5 | Health system | West |  |  |  | 1 | Yes |
| 6 | Practice | South | Yes | Yes | Rural | 1 | Yes |
| 7 | Health system | Northeast |  |  |  | 1 | Yes |
| 9 | Practice | West | Yes | No | Suburban | 1 | Yes |
| 11 | Health system | Northeast |  |  |  | 1 | Yes |
| 29 | Health system | South |  |  |  | 1 | Yes |
| 31 | Health system | Northeast |  |  |  | 1 | No |
| 32 | Health system | Midwest |  |  |  | 1 | No |
| 4 | Health system | West |  |  |  | 2 | Yes |
| 8 | Health system | Northeast |  |  |  | 2 | Yes |
| 10 | Practice | West | Yes | No | Urban | 2 | Yes |
| 12 | Health system | Northeast |  |  |  | 2 | Yes |
| 13 | Practice | Northeast | No | Yes | Rural | 2 | Yes |
| 15 | Practice | West | Yes | No | Suburban | 2 | Yes |
| 23 | Health system | Northeast |  |  |  | 2 | Yes |
| 30 | Practice | Northeast | Yes | Yes | Urban | 2 | No |
| 33 | Health system | West |  |  |  | 2 | No |
| 34 | Health system | South |  |  |  | 2 | No |
| 35 | Health system | West |  |  |  | 2 | No |
| 36 | Health system | Midwest |  |  |  | 2 | No |
| 37 | Health system | South |  |  |  | 2 | No |
| 14 | Practice | South | Yes | Yes | Suburban | 3 | Yes |
| 19 | Health system | Midwest |  |  |  | 3 | Yes |
| 21 | Practice | West | Yes | Yes | Suburban | 3 | Yes |
| 22 | Health system | Northeast |  |  |  | 3 | Yes |
| 25 | Health system | South |  |  |  | 3 | Yes |
| 38 | Health system | Midwest |  |  |  | 3 | No |
| 39 | Health system | South |  |  |  | 3 | No |
| 40 | Health system | Northeast |  |  |  | 3 | No |
| 41 | Health system | South |  |  |  | 3 | No |
| 16 | Practice | West | No | Yes | Urban | 4 | Yes |
| 17 | Practice | Midwest | Yes | Yes | Rural | 4 | Yes |
| 18 | Practice | West | Yes | Yes | Rural | 4 | Yes |
| 20 | Health system | Northeast |  |  |  | 4 | Yes |
| 24 | Health system | Midwest |  |  |  | 4 | Yes |
| 42 | Practice | Northeast | Yes | Yes | Urban | 4 | No |
| 43 | Practice | South | Yes | Yes | Rural | 4 | No |
| 44 | Practice | West | No | No | Urban | 4 | No |
| 45 | Practice | South | Yes | No | Multiple | 4 | No |
| 46 | Practice | South | Yes | Yes | Multiple | 4 | No |
| 47 | Health system | South |  |  |  | 4 | No |
| 48 | Health system | Midwest |  |  |  | 4 | No |
| 49 | Health system | South |  |  |  | 4 | No |
| 50 | Health system | West |  |  |  | 4 | No |
| 51 | Health system | West |  |  |  | 4 | No |
| 26 | Practice | South | Yes | Yes | Urban | 5 | Yes |
| 27 | Practice | Midwest | Yes | Yes | Urban | 5 | Yes |
| 52 | Practice | South | No | Yes | Suburban | 5 | No |
| 53 | Practice | West | Yes | Yes | Multiple | 5 | No |
| 54 | Practice | Northeast | No | Yes | Urban | 5 | No |
| 55 | Practice | Northeast | No | Yes | Urban | 5 | No |
| 56 | Practice | South | Yes | Yes | Suburban | 5 | No |
| 61 | Practice | Northeast | Yes | Yes | Suburban | 5 | No |
| 62 | Practice | West | No | Yes | Multiple | 5 | No |
| 28 | Practice | Northeast | No | Yes | Rural | 6 | Yes |
| 57 | Practice | Midwest | No | Yes | Suburban | 6 | No |
| 58 | Practice | South | No | Yes | Urban | 6 | No |
| 59 | Practice | West | No | Yes | Suburban | 6 | No |
| 60 | Practice | Midwest | No | Yes | Rural | 6 | No |
| 63 | Health system | South |  |  |  | 6 | No |
| 64 | Health system | West |  |  |  | 6 | No |

*Outreach was conducted in rolling waves with each wave being dependent upon the organization that agreed to participate from earlier waves. The number and type of organizations in each wave were selected to help provide a robust and diverse sample.

** We did not collect data on if a system could be designated as predominately safety net.

***Urbanicity and specialty mix are only reported for practice as most systems have both primary and specialty care, and span levels of urbanicity.

Appendix Table 3: Summary of participating and non-participating organizations

|  | **Participating organizations (n=29)** | **Non-participating organizations* (n=35)** |
| --- | --- | --- |
| **Organizational type** |  |  |
| Health system | 14 (48.3%) | 18 (51.4%) |
| Practice | 15 (51.7%) | 17 (48.6%) |
| **Region** |  |  |
| Midwest | 5 (17.2%) | 6 (17.1%) |
| Northeast | 10 (34.5%) | 7 (20.0%) |
| South | 5 (17.2%) | 13 (37.1%) |
| West | 9 (31.0%) | 9 (25.7%) |
| **Practice specialty mix**** |  |  |
| Primary care only | 12 (80.0%) | 15 (88.2%) |
| Multi-specialty | 3 (20.0%) | 2 (11.8%) |
| **Safety net practice***** |  |  |
| Yes | 11 (73.3%) | 8 (47.1%) |
| No | 4 (26.7%) | 9 (52.3%) |
| **Practice urbanicity**** |  |  |
| Urban | 5 (33.3%) | 6 (35.3%) |
| Suburban | 4 (26.7%) | 5 (29.4%) |
| Rural | 5 (33.3%) | 2 (11.8%) |
| Multiple | 1 (6.7%) | 4 (23.5%) |

*These organizations were contacted and asked to participate in this study, but they did not respond to outreach.

**Urbanicity and specialty mix are only reported for practice as we expect most systems to have both primary and specialty care and to span levels of urbanicity.

***We did not collect data on if a system included any safety net providers.

Appendix Table 4: Characteristics of participating organizations

| **Site** | **Description** | **Composition** | **Interviews** | **Interviewee(s) Role** | **Reason for second interview** |
| --- | --- | --- | --- | --- | --- |
| 1 | Urban family medicine clinic in the Midwest (10 to 20 providers) | Single primary care delivery site | 1 | Program Management |  |
| 2 | Health system in the Northeast | Hospital, primary care and specialty delivery sites | 1 | Program Management (2) |  |
| 3 | Coalition of community health centers in the West | Primary care clinics | 1 | Executive Leadership, Program Management |  |
| 4 | Health system in the West | Hospitals, primary care and specialty delivery sites | 1 | Executive Leadership |  |
| 5 | Health system in the West | Hospitals, primary care and specialty delivery sites | 1 | Executive Leadership |  |
| 6 | Rural FQHC in an area that covers two states in the South (1 to 10 providers) | Single primary care delivery site | 1 | Executive Leadership |  |
| 7 | Health system in the Northeast | Hospitals, primary care and specialty delivery sites | 1 | Executive Leadership, Program Management (2) |  |
| 8 | Rural healthcare system that includes hospitals in two states in the Northeast | Hospitals, primary care and specialty delivery sites | 2 | Executive Leadership, Program Management/Practicing Clinician | First interviewee suggested that the second interviewee may have additional insight as a practicing clinician involved in program implementation |
| 9 | Suburban FQHC with multiple clinical delivery sites in the West | Primary and specialty care delivery sites | 1 | Program Management |  |
| 10 | Urban FQHC with multiple locations in the West | Primary and specialty care delivery sites | 1 | Program Management |  |
| 11 | Accountable care organization in the Northeast | Hospitals, primary care and specialty delivery sites | 1 | Executive Leadership |  |
| 12 | Large health system in the Northeast that also manages its own health plan | Hospitals, primary care and specialty delivery sites; health plan | 2 | Executive Leadership, Case Management Staff | First interview discussed program goals across the system, secondary interview provided details on the case management process |
| 13 | Small rural practice in the Northeast | Single primary care delivery site | 1 | Program Management (2), Practicing Clinician |  |
| 14 | Large suburban FQHC in the South | Primary care delivery sites | 1 | Executive Leadership, Program Management, Case Management Staff |  |
| 15 | FQHC suburban in the West | Primary and specialty care delivery sites | 1 | Program Management |  |
| 16 | Urban practice in the West (less than 10 providers) | Single primary care delivery site | 1 | Case Management Staff |  |
| 17 | Rural community health center in the Midwest (20 to 40 providers) | Primary care delivery sites | 1 | Case Management Staff |  |
| 18 | Rural community health center in the West | Primary care delivery sites | 1 | Program Management |  |
| 19 | Health system in the Midwest | Hospitals, primary care and specialty delivery sites | 1 | Program Management (2) |  |
| 20 | Urban system in the Northeast | Hospitals, primary care and specialty delivery sites | 1 | Executive Leadership, Program Management |  |
| 21 | Suburban community health center in the West | Primary care delivery sites | 2 | Executive Leadership, Program Management | First interviewee suggested that second interviewee would be able to explain details on implementation of the programming within clinics |
| 22 | Health system in the Northeast | Hospitals, primary care and specialty delivery sites | 1 | Executive Leadership |  |
| 23 | Health system in the Northeast | Hospitals, primary care and specialty delivery sites | 2 | Program Management |  |
| 24 | Health system in the Midwest | Hospitals, primary care and specialty delivery sites | 1 | Executive Leadership, Program Management (2) | First interviewee provided an overview of broad strategic goals, secondary interviewee explained the details of program design and implementation |
| 25 | Health system in the South | Hospitals, primary care and specialty delivery sites | 1 | Program Management |  |
| 26 | Urban community health center in the South | Primary care delivery sites | 1 | Executive Leadership |  |
| 27 | Urban FQHC in the Midwest (20 to 50 providers) | Single primary care delivery site | 1 | Executive Leadership |  |
| 28 | Rural independent practice in the Northeast (less than 10 providers) | Single primary care delivery site | 1 | Practicing Clinician |  |
| 29 | Urban academic health system in the South | Hospitals, primary care and specialty delivery sites | 1 | Executive Leadership, Program Management |  |
