# Supplementary material for: A Missed Opportunity? How Health Care Organizations Engage Primary Care Clinicians in Formal Social Care Efforts
Source: Popul Health Manag. 2022 Aug 8;25(4):509–16. doi: 10.1089/pop.2021.0306 (PMC9419929; doi:10.1089/pop.2021.0306)
Supplement: Supplemental data [file Suppl_AppendixFigS1.docx]

Appendix Figure 1: Analytic Approach
