# Supplementary material for: A Missed Opportunity? How Health Care Organizations Engage Primary Care Clinicians in Formal Social Care Efforts
Source: Popul Health Manag. 2022 Aug 8;25(4):509–16. doi: 10.1089/pop.2021.0306 (PMC9419929; doi:10.1089/pop.2021.0306)
Supplement: Supplemental data [file Suppl_AppendixTableS1.docx]

**Appendix**

Appendix Table 1: Interviewee Categories

| **Interviewee Categories** | **Description** | **Examples** |
| --- | --- | --- |
| Executive Leadership | Individuals primarily responsible for overseeing the operations of the entire organization | Chief Executive Officer, Chief Clinical Officer |
| Program Management | Individuals who oversee specific departments or services | Program Manager, Community Relations Manager, Eligibility Supervisor |
| Case Management Staff | Individuals who worked within case management teams and who focused primarily on case management activities | Nurse Care Manager, Community Health Worker, Social Worker, Navigator |
| Practicing Clinician | Individuals whose primary role was the provision of medical care | Physician |
