# Supplementary material for: A Missed Opportunity? How Health Care Organizations Engage Primary Care Clinicians in Formal Social Care Efforts
Source: Popul Health Manag. 2022 Aug 8;25(4):509–16. doi: 10.1089/pop.2021.0306 (PMC9419929; doi:10.1089/pop.2021.0306)
Supplement: Supplemental data [file Suppl_AppendixTableS5.docx]

Appendix Table 5: Interview Guide Domains

| **Domain** | **Sub-domains** |
| --- | --- |
| Organizational Characteristics | - Organization size and structure - Interviewee role - Motivations for social needs work - Populations served - Participation in delivery reforms |
| Screening | - Which patients screened - Needs screened for - Screening workflows, methods, tools used - Staff involved with screening - Follow-up processes - Access to screening results - Frequency of screening - Reason for starting screening - Development of screening program - Engagement with clinicians - Buy-in from staff - Plans for scaling, changing screening program - Common needs patients have |
| Referrals | - Workflow - Staff involved - Tailoring to patients - Variation between locations, patients - Referral lists, referral platforms   - Development   - Maintenance   - Staff involved   - Tracking use of referrals - Role of clinicians - Buy-in from clinicians and patients - Follow-up processes - Closed loop referrals - Common challenges with referrals |
| Assistance | - Workflow - Types of assistance offered - Staff involved - Staff training - Variation between patients - Engagement with community organizations - Communication with   - Patients   - Clinicians   - Other staff - Centralized vs. decentralized programs - Tracking of patients/data collection - Common challenges - Reason for starting assistance work - Program development - Changes made to program |
| Need specific programming (e.g., food, housing, transportation) | - Internal programs - External programs - Funding - Services - Types of patients - Development processes |
| Interactions with community-based organizations (CBOs) | - Types of partners - Role of:   - Health care organization   - CBO - History of partnerships - Involvement of CBO in program development - Formalized or ad-hoc - Contractual relationships - Types of patients served - Data/records sharing |
| Overview/Reflection | - Challenges faced - Challenges solved - Overlap with care management - Organizational buy-in - Advice for other organizations - Organizational goals - Needed support (financial, resources, policy) |
